# RUNX1 Is Regulated by Androgen Receptor to Promote Cancer Stem Markers and Chemotherapy Resistance in Triple Negative Breast Cancer

**DOI:** 10.3390/cells12030444

**Published:** 2023-01-29

**Authors:** Natalia B. Fernández, Sofía M. Sosa, Justin T. Roberts, María S. Recouvreux, Luciana Rocha-Viegas, Jessica L. Christenson, Nicole S. Spoelstra, Facundo L. Couto, Ana R. Raimondi, Jennifer K. Richer, Natalia Rubinstein

**Affiliations:** 1 Instituto de Biociencias, Biotecnología y Biología Traslacional (iB3), Departamento de Fisiología, Biología Molecular y Celular, Facultad de Ciencias Exactas y Naturales, Universidad de Buenos Aires, Buenos Aires C1428EGA, Argentina; 2 Consejo Nacional de Investigaciones Científicas y Técnicas (CONICET), Godoy Cruz 2290, Buenos Aires C1425FQB, Argentina; 3 Department of Pharmacology, University of South Alabama College of Medicine, Mobile, AL 36688, USA; 4 Department of Obstetrics and Gynecology, David Geffen School of Medicine, University of California Los Angeles, Los Angeles, CA 90095, USA; 5 Instituto de Fisiología, Biología Molecular y Neurociencias (IFIBYNE-UBA-CONICET), Consejo Nacional de Investigaciones Científicas y Técnicas de Argentina-Universidad de Buenos Aires, Buenos Aires C1428EHA, Argentina; 6 Department of Pathology, Anschutz Medical Campus, University of Colorado, Aurora, CO 80045, USA

**Keywords:** RUNX1, AR, CSC, chemoresistance, TNBC

## Abstract

Triple negative breast cancer (TNBC) is an aggressive breast cancer subtype for which no effective targeted therapies are available. Growing evidence suggests that chemotherapy-resistant cancer cells with stem-like properties (CSC) may repopulate the tumor. The androgen receptor (AR) is expressed in up to 50% of TNBCs, and AR inhibition decreases CSC and tumor initiation. Runt-related transcription factor 1 (RUNX1) correlates with poor prognosis in TNBC and is regulated by the AR in prostate cancer. Our group has shown that RUNX1 promotes TNBC cell migration and regulates tumor gene expression. We hypothesized that RUNX1 is regulated by the AR and that both may work together in TNBC CSC to promote disease recurrence following chemotherapy. Chromatin immunoprecipitation sequencing (ChIP-seq) experiments in MDA-MB-453 revealed AR binding to *RUNX1* regulatory regions. RUNX1 expression is upregulated by dihydrotestosterone (DHT) in MDA-MB-453 and in an AR^+^-TNBC HCI-009 patient-derived xenograft (PDX) tumors (*p* < 0.05). RUNX1 is increased in a CSC-like experimental model in MDA-MB-453 and SUM-159PT cells (*p* < 0.05). Inhibition of RUNX1 transcriptional activity reduced the expression of CSC markers. Interestingly, RUNX1 inhibition reduced cell viability and enhanced paclitaxel and enzalutamide sensitivity. Targeting RUNX1 may be an attractive strategy to potentiate the anti-tumor effects of AR inhibition, specifically in the slow-growing CSC-like populations that resist chemotherapy which lead to metastatic disease.

## 1. Introduction

Triple negative breast cancer (TNBC) is a heterogenous disease that includes all breast cancer subtypes that have no expression of estrogen and progesterone receptors, nor amplification of HER2 [1]. Accounting for 15–20% of all breast cancers, TNBC is more prevalent in younger women and women of African and Hispanic descents, as reviewed in Zagami et al. (2022) [2]. TNBC has recently been subdivided into four subtypes on the basis of gene expression profiles: basal-like immunosuppressed (BLIS), immunomodulatory (IM), luminal androgen receptor (LAR), and mesenchymal (MES) [3]. Despite major advances in breast cancer treatments, the heterogeneity of TNBC is still a challenge, since there are few targeted therapy options, leaving surgery, chemotherapy, and radiation as the first line of treatment [4]. Unfortunately, 35% of tumors will evade these strategies, leading to a high rate of rapid recurrence as metastatic disease [5]. The lack of higher-precision therapeutic targets is an unmet need for these tumors and contributes to the more aggressive behavior of this tumor subtype [4,6,7]. Studies suggest that chemotherapy-resistant cancer cells with stem-like properties may repopulate the tumor locally or cause recurrence as metastatic disease [4,8,9,10]. Thus, therapies that target the cancer stem cell (CSC)-like population in combination with chemotherapy to prevent rapidly dividing cells may impair tumor recurrence. Growing evidence suggests that chemotherapy resistance is associated with the more slowly dividing CSC subpopulation [4,8,9,10].

The LAR subtype represents 20–40% of TNBCs and is characterized by expression of the androgen receptor (AR), a luminal-like pattern of gene expression [3], as well as reduced pathologic complete response (pCR) to neoadjuvant chemotherapy [11]. Moreover, accumulating preclinical evidence suggests that as many as half of TNBC, not just LAR subtype, may be dependent on AR [12,13]. Furthermore, AR has emerged as a potential therapeutic target in breast cancer and its efficacy in AR^+^-TNBC (Stage I–III) patients is currently under evaluation, based on a protocol with enzalutamide (Enza) and paclitaxel (Px) before surgery (clinical trial NCT02689427). Indeed, AR inhibition significantly reduces baseline proliferation, anchorage-independent growth, migration, and invasion, and increases apoptosis in AR^+^-TNBC lines [12]. In vivo, Enza significantly decreases viability of SUM-159PT and HCC1806 xenografts [12]. Moreover, the AR supports CSC-like properties, including anchorage-independent survival and mammosphere formation [14]. Pretreatment with Enza reduces tumor volume and viability when administered simultaneously or subsequently with Px, where simultaneous treatment suppressed tumor recurrence more effectively in mice after drug cessation [14]. Moving forward, a biomarker for the selection of patients with TNBC suitable for treatment with AR inhibitors is a major unmet need [7]. Nevertheless, a precise understanding of the mechanism of androgen action in this disease remains a challenge.

The Runt-related transcription factor (RUNX) family regulates a plethora of developmental processes, including cell growth, differentiation, and lineage specification [15,16,17]. During mammary development, RUNX factors are important for the maintenance of mammary epithelium homeostasis [18,19]. In human breast cancer, RUNX1 activity is still a matter of debate and little is known about its role in tumor progression. Accumulating evidence strongly suggests that RUNX1 promotes tumor aggressiveness in TNBC, while functioning as a tumor suppressor in ER^+^ breast cancer [18,20,21,22,23,24,25,26,27].

According to Cancer Genome Atlas Network (2012) and the analysis performed by Caldas Group [28], the *RUNX1* gene is mutated only in Luminal A/B tumors. Moreover, in the Catalogue of Somatic Mutations in Cancer, RUNX1 is included in the top 20 mutated genes. However, only a few mutations have been reported in TNBC samples suggesting that it might be relevant to maintain wild-type protein expression in some TNBC subgroups (COSMIC, http://cancer.sanger.ac.uk/cosmic/). Additionally, RUNX1 has an independent prognostic indicator of poor patient outcomes in TNBC [21]. RUNX transcription factors and their coregulator, core binding factor beta CBFβ, promote phenotypic plasticity and are essential for maintaining the mesenchymal and invasive phenotype [24]. Our group showed that RUNX1 regulates R-Spondin 3 (RSPO3), promoting tumor growth and motility [22,29]. Strikingly, it has been reported that RUNX1 is involved in the differentiation or reduction of normal and tumoral ER^+^ mammary stem cells [25,30,31] and in the proliferation of mesenchymal prostate stem cell proliferation [32]. However, there is no data on TNBC-CSC as of yet. While AR-mediated activation of RUNX1 has previously been reported in prostate cancer cells, evidence for the AR directly binding to the RUNX1 promoter/gene body is limited in that context and has not been demonstrated in breast cancer [33].

Our goal was to investigate the relevance of RUNX1 in AR^+^-TNBC tumors. Our hypothesis is that RUNX1 is regulated by AR activation to promote CSC enrichment, leading to a chemoresistant population capable of surviving and metastasizing to distant organs. AR activation by dihydrotestosterone (DHT) induces *RUNX1* gene expression in vitro and in vivo in AR^+^-TNBC PDX tumor samples. By inhibiting RUNX1 transcriptional activity we determined its requirement to induce CSC genes and to enhance chemoresistance. Our results show, for the first time, that the AR induces RUNX1 expression in TNBC cell lines, promoting CSC phenotype enrichment and increased chemoresistance. Furthermore, these data suggest that the AR and RUNX1 might work together to promote tumor progression, and to be useful for clinical therapeutic decision-making in AR^+^-TNBC.

## 2. Materials and Methods

### 2.1. TNBC Cell Lines and Reagents

All cell lines used in this study are AR^+^-TNBC [13]. MDA-MB-453 (luminal androgen receptor, LAR) cells were purchased from the ATCC and maintained in DMEM high glucose medium (Gibco; Thermo Fisher Scientific, Waltham, MA, USA) with 10% fetal bovine serum (FBS, Sigma-Aldrich; Merck, Darmstadt, Germany). SUM-159PT (mesenchymal stem-like, MSL) cells were obtained from the University of Colorado Cancer Center (UCCC) Tissue Culture Core (Aurora, CO) and maintained in Ham’s F-12 (Gibco) with 5% FBS, 1% HEPES (Gibco), 1 μg/mL hydrocortisone (Sigma-Aldrich), and 5 μg/mL insulin (Sigma-Aldrich). BT-549 (mesenchymal-like, ML) cells, purchased from the ATCC, were grown in RPMI 1640 medium (Gibco) with 10% FBS, nonessential amino acids (NEAA, Gibco), and 5 μg/mL insulin supplementation. MDA-MB-231 (mesenchymal stem-like, MSL) cells were grown in RPMI 1640 medium with 10% FBS. All cells were grown in the presence of 1% streptomycin and amphotericin B (Gibco) and maintained at 37 °C in a humidified incubator containing 95% air and 5% CO_2_. Cell lines were authenticated by short tandem repeat DNA profiling (Promega, Madison, WI, USA) at the UCCC Cell Technologies Shared Resource (September 2020). Mycoplasma testing was performed at the University of Colorado and University of Buenos Aires regularly.

Dihydrotestosterone (DHT, Sigma-Aldrich) was diluted in ethanol, enzalutamide (Enza, Sigma-Aldrich; #PHB00235) in DMSO, Paclitaxel (Px, Cell Signaling Technology, Danvers, MA, USA; #98075) in DMSO and RUNX1 inhibitors AI-10-104 (Glixx Laboratories, Hopkinton, MA, USA; GLXC-20705) and AI-10-49 (Glixx Laboratories GLXC-07203) in DMSO.

All experiments that included DHT and/or enzalutamide treatment were conducted in charcoal-stripped serum.

### 2.2. Forced Suspension Culture

Poly-2-hydroxyethyl methacrylate (poly-HEMA, Sigma-Aldrich) was prepared at a concentration of 12 mg/mL in 95% ethanol. Culture plates were incubated with poly-HEMA overnight to allow ethanol evaporation. Plates were washed with PBS prior to use.

### 2.3. Quantitative RT-PCR

RNA was isolated by TRI reagent (MRC, Cincinnati, OH, USA) and cDNA was synthesized from 1 ug total RNA, using M-MLV reverse transcriptase enzyme (Promega). SYBR Green quantitative gene expression analysis was performed using Taq polymerase (Invitrogen, Thermo Fisher Scientific) in a StepOne instrument (Applied Biosystem, Thermo Fisher Scientific). Relative gene expression was calculated using the 2^–∆∆Ct^ method and values were normalized to GAPDH. Primer sequences are listed in supplementary Table S4a.

### 2.4. Western Blot

Protein extracts were prepared in a cell lysis buffer and denatured at 95 °C for 10 min, separated on SDS-PAGE gels and transferred to nitrocellulose membranes (Bio-rad Laboratories, Hercules, CA, USA). After blocking in 5% non-fat milk in T-TBS, membranes were incubated overnight at 4 °C with primary antibodies in 0.5% BSA or 2% non-fat milk in T-TBS: anti-AR (1:1000 dilution; Santa Cruz Biotechnology, Dallas, TX, USA; #7305), anti-RUNX1 (1:1000; Cell Signaling Technology; #4334), anti-SOX4 (1:1000; Abcam #52043), anti-Tubulin (1:10,000; Sigma-Aldrich; #T5168), and anti-GAPDH (1:5000; Santa Cruz Biotechnology; #32233). Secondary antibody incubation was performed at room temperature for 1 h: anti-mouse (1:5000; Li-Cor #926-32213 or Li-Cor #926-68070) or anti-rabbit (1:5000; Li-Cor; Lincoln, NE, USA; #926-32210). Membranes were then scanned using the Odyssey Imaging System and analyzed with Image Studio Ver 5.2 software (Li-Cor). Quantifications are presented below each blot as the relative mean of all experiments performed plus/minus the standard deviation, normalized to the housekeeping. All experiments were performed at least three times.

### 2.5. Chromatin Immunoprecipitation

For AR ChIP-seq, MDA-MB-453 cells were grown in charcoal-stripped serum media for a total of 72 h before treatment. Twenty-four hours prior to treatment, cells were trypsinized and equal cell numbers were plated on control tissue culture dishes (attached) or poly-HEMA coated dishes (suspended). Cells were treated with DMSO (vehicle control), DHT (10 nM), or DHT + Enza (10 µM) for 4 h, followed by fixation in 1% formaldehyde. ChIP-seq was performed as previously reported [34]. Chromatin was sonicated using an EpiShear Probe Sonicator (Active Motif, Carlsbad, CA, USA) for 4 min (cycles of 30 s with 30 s of rest in between) at 40% power. AR antibody H-280 (Santa Cruz Biotechnology) was utilized for immunoprecipitation and libraries were sequenced on an HiSeq 2500 (Illumina, San Diego, CA, USA).

RUNX1 ChIP in an MDA-MB-231 cell line was performed as previously described [22] using anti-RUNX1 (Abcam, Cambridge, UK; #23980) and anti-IgG (Abcam; #46540, negative control). Primer sequences are available in supplementary Table S4b.

### 2.6. ALDEFLUOR

The ALDEFLUOR assay (Stem Cell Technologies, Vancouver, BC, Canada) was performed per the manufacturer’s protocol and as previously reported [14]. Briefly, 1.2 × 10^7^ cells/plate (15 cm poly-HEMA coated plates) were grown in forced-suspension for 3 days. ALDEFLUOR-positive and -negative cell populations were sorted with the assistance of the UCCC Flow Cytometry Shared Resource on the MoFlo XDP100 cell sorter (Beckman Coulter Life Sciences, Indianapolis, IN, USA). Immediately after sorting, mRNA from the two ALDH subpopulations was prepared and qRT-PCR was performed.

### 2.7. Crystal Violet

Cells were plated in 96-well plates in quadruplicate or quintuplicate and treated with increasing concentrations of AI-10-104/-49 (for dose-response curves) or with Px, Enza, and AI-10-104/-49 alone or in combination (for cytotoxicity assays). After 3 days of drug treatments, cells were fixed with 10% formalin, stained with 0.1% crystal violet, and then solubilized with 10% acetic acid. Absorbance was measured at 540 nm. In parallel, another plate with an increasing number of cells was prepared to generate a calibration curve. Data are presented as a percentage of cell viability relative to control treated cells (vehicle, DMSO).

### 2.8. MTT

Cells were plated in 96-well plates in quintuplicate and treated with Px, Enza, and AI-10-104/-49 alone or in combination. After 3 days of drug treatments, 0.5 μg/ul thiazolyl blue tetrazolium bromide (Sigma-Aldrich; #M5655) was added at each well and incubated for 4 h at 37 °C. Next, 0.01N Isopropanol was used to dissolve the formazan crystals and absorbance at 570 nm was measured. Data are presented as the relative absorbance to control treated cells (vehicle, DMSO).

### 2.9. Statistical Analysis

Statistical significance was evaluated using two-tailed unpaired Student *t*-tests or one/two-way ANOVA followed by Tukey contrast with GraphPad Prism 9 software (Dotmatics, Bishop’s Stortford, UK). All experiments were performed at least 3 times before analyzing the statistical significance. A *p* value of less than 0.05 was considered statistically significant.

## 3. Results

### 3.1. Androgen Receptor Regulates RUNX1 Gene Expression

**Figure 1:**
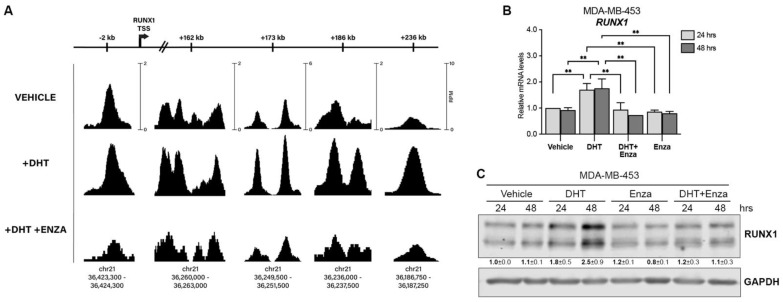
AR regulates RUNX1 expression. (**A**) MDA-MB-453 cells were treated with either vehicle (DMSO + ethanol), DHT (10 nM) or DHT + Enza (20 μM) for 24 h. AR ChIP-seq was performed and the *RUNX1* promoter and intronic regions were analyzed. The scale bars at the top are labeled for each locus to indicate the relative distance from the canonical RUNX1 transcription start site (TSS). The AR ChIP-seq signals are shown for each treatment condition at multiple loci within RUNX1. The scale at each position (shown in the ‘VEHICLE’ track) indicates the normalized range of signal values within that region (as measured by reads per million, RPM) and is the same for all treatment conditions to allow for accurate comparison of the peak intensities. The chromosomal coordinates of each peak are shown below. RUNX1 qPCR (**B**) and Western blot (**C**) were performed in MDA-MB-453 cells treated with either vehicle (DMSO + ethanol), DHT (10 nM), Enza (20 μM), or both for 24 and 48 h. GAPDH was used as a housekeeping control. Two-way ANOVA followed by Tukey multiple comparison was performed in (**B**) from three independent experiments, ** *p* < 0.01. One representative experiment of three is shown in (**C**), mean ± SEM is indicated.

To evaluate the capacity of the androgen receptor (AR) to regulate *RUNX1* gene expression, an AR ChIP-seq assay was performed in MDA-MB-453, a representative LAR TNBC cell line according to the gene expression profile [35,36,37]. AR binds to the *RUNX1* promoter and four intronic loci within the *RUNX1* gene body (Figure 1A). Treatment for 24 h with DHT (an AR agonist) increases AR recruitment to *RUNX1* in all sites; this effect was blocked in the presence of the AR antagonist Enza. Moreover, DHT treatment upregulates *RUNX1* mRNA (Figure 1B) and protein (Figure 1C) levels, an effect that is also blocked when Enza is added.

This modulation is also observed in RNA-seq of HCI-009, an AR^+^ PDX tumor grown in mice with or without DHT, which showed significant upregulation of RUNX1 (fold change = 1.84, *p* = 1.27 × 10^−3^ [13]). Furthermore, scRNA-seq analysis between AR^High^ and AR^Low^ MDA-MB-453 cell populations showed significantly higher *RUNX1* expression in the AR^High^ cells (fold change = 1.53, *p* = 3.20 × 10^−12^ [13]). Together these data show that the AR positively regulates RUNX1 in AR^+^-TNBC.

### 3.2. RUNX1 Is Upregulated in a Circulating Tumor Cell Model and Contributes to the CSC Phenotype

**Figure 2:**
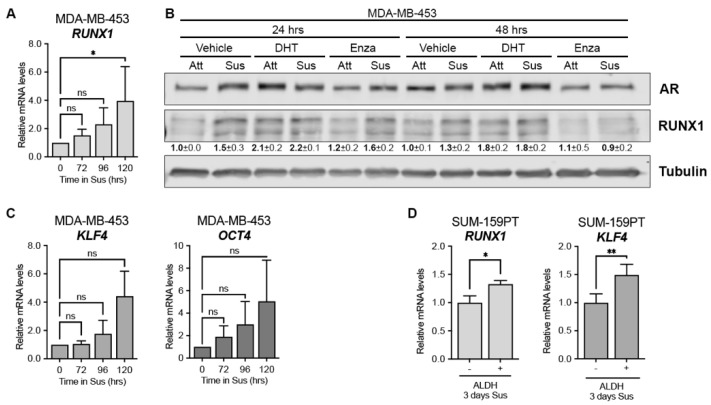
RUNX1 is upregulated in CSC-like subpopulations. mRNA levels of *RUNX1* (**A**), *KLF4*, and *OCT4* (**C**) after 72, 96 and 120 h in forced suspension (Sus) were determined by qPCR. *GAPDH* was used as a housekeeping control. (**B**) AR and RUNX1 Western blot of MDA-MB-453 cells culture in attached (Att) or forced-suspension conditions (Sus) and treated with either vehicle (DMSO + ethanol), DHT (10 nM) or Enza (20 μM) for 24 and 48 h. Tubulin was used as a housekeeping control. (**D**) SUM-159PT were cultured in forced-suspension conditions for 3 days and then sorted using ALDEFLUOR assay. mRNA was prepared from ALDH^−^ and ALDH^+^ subpopulations and RUNX1 and KLF4 levels were evaluated by qPCR. *GAPDH* was used as a housekeeping control. One-way ANOVA followed by Tukey multiple comparison (**A**,**C**) or Student *t*-test (**D**) were performed from three independent experiments. * *p* < 0.05, ** *p* < 0.01 and ns = not significant. One representative experiment of three is shown in (**C**), mean ± SEM is indicated.

Circulating tumor cells (CTC) with a CSC-like phenotype have been described as the major source of clinical tumor recurrence [38,39]. To evaluate the contribution of RUNX1 in the physiology of this subpopulation, we used a forced suspension in vitro cell culture model, since AR^+^-TNBC cells grown in these conditions’-express higher levels of AR and CSC markers such as CD24/CD44, and increased ALDH activity levels than their attached counterparts [14]. We observed that the AR binding sites in the *RUNX1* gene were conserved when MDA-MB-453 cells were cultured in forced suspended conditions and responded to DHT and Enza treatment (Figure S1A), as in attached conditions. We found that *RUNX1* mRNA is upregulated in MDA-MB-453 cells surviving in forced suspension culture over time (Figure 2A) and that treatment with DHT and Enza also modulates RUNX1 protein levels in these conditions (Figure 2B and Figure S1B). The rise of RUNX1 expression in this CTC model was accompanied by an increase of CSC markers, such as krüppel-like factor 4 (*KLF4*) and octamer-binding transcription factor 4 (*OCT4*) (Figure 2C).

Since non-LAR TNBC also critically depend on AR [12] we expanded our study with an AR^+^ non-LAR cell line, SUM-159PT. It was previously reported that SUM-159PT cells cultured for 3 days in forced suspension increased the population of aldehyde dehydrogenase-positive (ALDH^+^) cells by 60% and expressed significantly higher AR levels [14]. Since ALDH is a CSC marker we evaluated RUNX1 expression in this ALDH subpopulation. ALDH^+^ cells express significantly higher levels of *RUNX1* and *KLF4* after 3 days in forced suspension compared to levels in ALDH^−^ cells, which is consistent with a CSC-like phenotype (Figure 2D). AR expression was examined as an internal positive control (Figure S1C).

**Figure 3:**
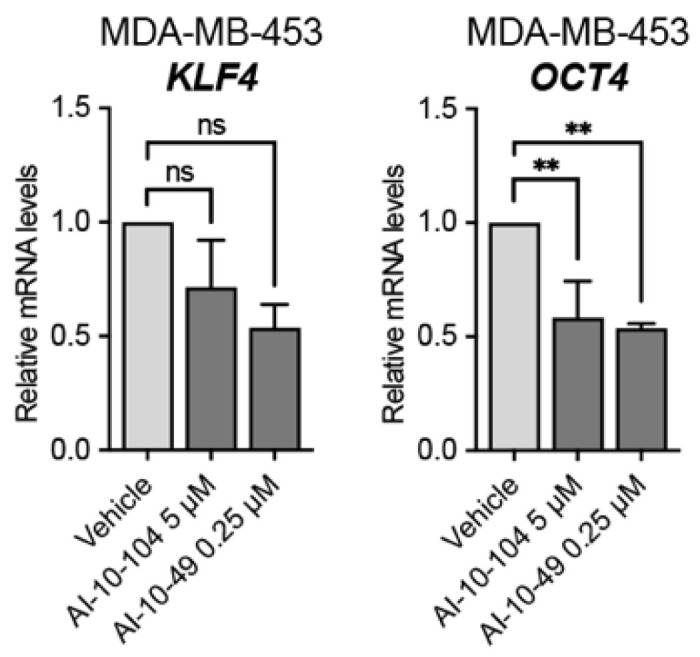
RUNX1 is required for the generation of a CSC-like phenotype. MDA-MB-453 cells were cultured in forced-suspension for 4 days and treated for one extra day with AI-10-104 (5 μM) or AI-10-49 (0.25 μM). mRNA levels of *KLF4* and *OCT4* were determined by qPCR. *GAPDH* was used as a housekeeping control. One-way ANOVA followed by Tukey multiple comparison was performed from three independent experiments, ** *p* < 0.01 and ns = not significant.

To investigate the contribution of RUNX1 in modulating the CSC phenotype in this CTC model we inhibited its transcriptional activity and measured the expression of CSC gene markers. RUNX1 commercial inhibitors AI-10-104 and -49 both downregulated *KLF4* and *OCT4* gene expression in MDA-MB-453 cells cultured in forced suspension (Figure 3).

**Figure 4:**
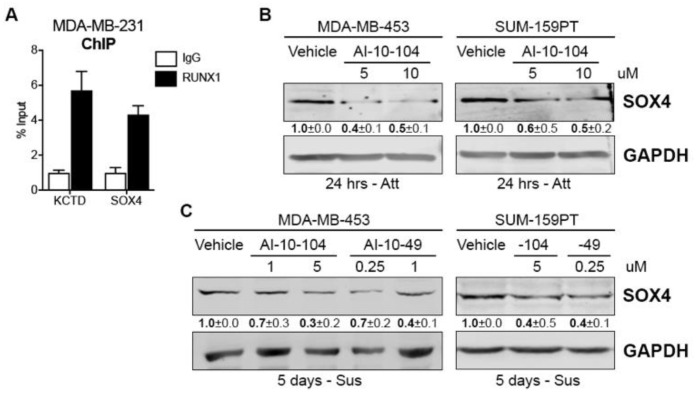
RUNX1 binds to the *SOX4* promoter and regulates its expression (**A**) RUNX1 ChIP assay was performed in MDA-MB-231 and *SOX4* promoter primers were designed to determine RUNX1 binding by qPCR. *KCTD* was used as a positive control. (**B**) SOX4 Western blot was performed in MDA-MB-453 and SUM-159PT cultured in attached (Att) conditions and treated with AI-10-104 (5 or 10 μM) for 24 h. GADPH was used as a housekeeping control. (**C**) MDA-MB-453 and SUM-159PT were cultured in forced-suspension (Sus) for 4 days and treated for one extra day with AI-10-104 (1 or 5 μM) or AI-10-49 (0.25 or 1 μM). GADPH was used as a housekeeping control. One representative experiment of three is shown in (**B**,**C**), mean ± SEM is indicated.

To further investigate RUNX1 involvement in the CSC phenotype we examined other relevant putative target genes. A previous RUNX1 ChIP assay performed by our group in MDA-MB-231 cells revealed that RUNX1 binds to different tumor-related genes [22]. Here we report that RUNX1 also binds to the SOX4 gene promoter (Figure 4A). SOX4 is an interesting transcription factor because it has been implicated in breast cancer Epithelial-Mesenchymal Transition (EMT) [40], metastasis [41], and drug resistance in other tumors such as colon cancer [42]. To evaluate the ability of RUNX1 to regulate SOX4 expression, MDA-MB-453 and SUM-159PT cell lines were treated with AI-10-104 for 24 h. SOX4 protein is decreased in these cell lines by RUNX1 inhibition (Figure 4B). Moreover, when these cell lines were cultured in forced suspension for 4 days and treated with RUNX1 inhibitors for 24 h, SOX4 protein was also downregulated (Figure 4C).

Interestingly, *KLF4* and *SOX4* mRNAs were also upregulated in AR^High^ cells compared to AR^Low^ cells (fold change = 1.7, *p* value = 4.67 × 10^−14^ and fold change = 1.82, *p* value = 8.23 × 10^−51^, respectively [13]), suggesting that, even under standard adherent culture conditions, these genes might be involved in the AR/RUNX1 axis that drives tumor cell fate.

### 3.3. Loss of RUNX1 Transcriptional Activity Reduces AR^+^-TNBC Viability and Enhances Drug Sensitivity

**Figure 5:**
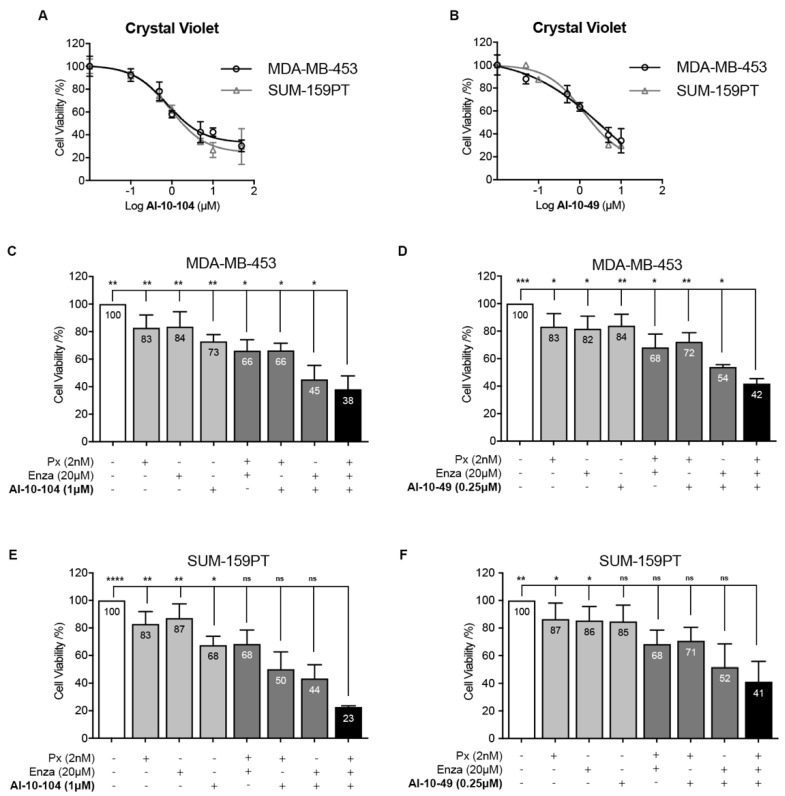
Inhibition of RUNX1 transcriptional activity reduces cell viability and enhances drugs’ cytotoxic effects. (**A**,**B**) MDA-MB-453 and SUM-159PT cells were cultured for 72 h and treated with increasing concentration of AI-10-104 (**A**) or AI-10-49 (**B**). MDA-MB-453 (**C**,**D**) and SUM-159PT (**E**,**F**) were treated with 2 nM Px, 20 μM Enza and 1 μM AI-10-104 (**C**,**E**) or 0.25 μM AI-10-49 (**D**,**F**) or all possible combinations for 72 h. In all cases, cell viability was determined by crystal violet assay (absorbance at 570 nm) using a calibration curve. Percentage of cell viability was calculated and expressed as relative to vehicle treatment (DMSO, 100%). Statistical differences are indicated in the graph only for the combination of Px, Enza, and AI-10-104 or -49 (black bars) vs. the rest of the treatments/vehicle. See Supplementary Tables S1 and S2 for the remaining statistical comparisons. One-way ANOVA followed by Tukey multiple comparison was performed from five independent experiments (**C**–**F**), * *p* < 0.05, ** *p* < 0.01, *** *p* < 0.001, **** *p* < 0.0001 and ns = not significant.

Since the CSC and EMT phenotypes are involved in TNBC drug resistance [4,8,9,10], we explored RUNX1 involvement in chemoresistance. To investigate the role of RUNX1 in response to chemotherapeutic drugs, AR^+^-TNBC cell lines were treated with the RUNX1 transcriptional activity inhibitors AI-10-104 and AI-10-49, the AR antagonist Enza, and the chemotherapeutic drug Px. It is known that AR inhibition combined with chemotherapy resulted in a more effective outcome than chemotherapy alone in vitro and in vivo in preclinical mouse models [12]. This combination is currently being tested in a clinical trial (NCT02689427) based on these preclinical data. Reduction in RUNX1 transcriptional activity decreases MDA-MB-453 and SUM-159PT cell viability in a dose dependent manner (Figure 5A,B). Inhibition of RUNX1 also reduces tumor cell colony formation (Suppl Figure S2A). Importantly, reduction of RUNX1 transcriptional activity with AI-10-104 or AI-10-49 significantly increased sensitivity to Px and Enza in standard tissue culture conditions after 72 h in MDA-MB-453 cells (Figure 5C,D). A similar effect was observed in SUM-159PT (Figure 5E,F) and BT-549 non-LAR TNBC cell lines (Figure S2B, MTT assay).

**Figure 6:**
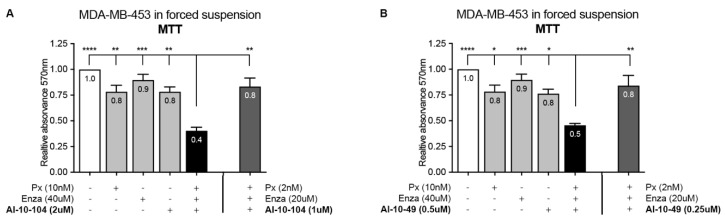
Reduction of RUNX1 transcriptional activity enhances enzalutamide and paclitaxel treatment in CSC-like cells. MDA-MB-453 cells were cultured for 72 h in forced-suspension and treated with either 10 nM Px, 40 μM Enza and 2 μM AI-10-104 (**A**)/0.5 μM AI-10-49 (**B**) or the doses used for attached conditions (dark gray bar): 2 nM Px, 20 μM Enza and 1 μM AI-10-104 (**A**)/0.25 μM AI-10-49 (**B**) for 72 h. Cell viability was determined by MTT and the results are expressed as the relative absorbance (570 nm) to control treatment (DMSO, 1.00). Statistical differences are shown for the combination of the 3 drugs (black bar) vs. the rest of the treatments (gray bars) or vehicle (white bar). For more details see Suppl Table S3. One-way ANOVA with Tukey multiple comparison was performed from three independent experiments, * *p* < 0.05, ** *p* < 0.01, *** *p* < 0.001, and **** *p* < 0.0001.

To further explore the role of RUNX1 in CSC/CTC drug resistance, anchorage-independent cells were treated with Enza, Px, and RUNX1 inhibitors for cell survival evaluation by MTT assay. A reduction in RUNX1 transcriptional activity significantly improves the efficacy of Px and Enza in forced suspension culture (Figure 6, compare black bar with light gray bars). Under these suspended conditions higher doses of all drugs were required, including RUNX1 inhibitors, to achieve a reduction in the cell viability rate similar to that obtained in standard adherent cultures (Figure 6, compare dark gray bar using attached condition drugs doses with black bar). This observation validates the predetermined concept that CSC populations are more resistant to treatment [43].

Taken together, these results strongly suggest that RUNX1 transcriptional activity is necessary for TNBC cells to survive chemotherapy. More importantly, they show that reducing RUNX1 transcriptional activity may be an opportunity to improve the sensitivity of CSC/CTC to chemotherapy leading to a potential reduction in metastasis or tumor recurrence in AR^+^-TNBC.

## 4. Discussion

Here we show for the first time that the AR is a direct positive regulator of *RUNX1* gene expression in AR^+^-TNBC and that RUNX1 transcriptional activity is involved in the CSC-like phenotype and chemoresistance, contributing to tumor progression in this aggressive breast cancer subtype.

One of the current principal clinical challenges in TNBC is the presence of inter- and intratumoral heterogeneity that hinders decision-making and drives the lack of therapeutic efficacy [44,45]. Therefore, identification of better markers could reveal more accurate therapeutic strategies. Although several findings support a role for the androgen/AR axis in breast cancer, its involvement in the pathogenesis and progression of this cancer remains under debate. The predictive and prognostic role of the AR in TNBC is still clinically undetermined [46]. Accumulating data suggests that, even if a protein is indicative of a more well-differentiated tumor, it can serve as a therapeutic target if tumors are dependent upon it for growth (as is well acknowledged for targeting ER). It is clear that LAR-TNBC has a poor pathological complete response (pCR) [11]; and anti-androgen therapy (to kill the slow-growing cells) combined with standard chemotherapy drugs, such as paclitaxel (to kill rapidly dividing tumor cells), showed promising results in AR^+^-TNBC preclinical models [12,14]. Interestingly, a clinical trial is currently underway in AR^+^-TNBC patients using this drug combination (NCT02689427). The determination of better biomarkers for selecting patients suitable for treatment with AR inhibitors is a major unmet need.

We determined that RUNX1 may be an appropriate marker for anti-androgen treatment both in LAR and non-LAR AR^+^-TNBC, as well a potential therapeutic target by itself. AI-10-104 and AI-10-49 are small molecules that bind to CBFβ and inhibit its binding to RUNX proteins impairing their transcriptional activity [47]. RUNX1 inhibition strategy is still at preclinical stage in cancer pathologies. The use of these inhibitors to study chemoresistance in TNBC is reported here for the first time, but other research have been conducted, including in vivo approaches in acute myeloid leukemia [48]. In particular, the toxicity of AI-10-49 has been tested in mice with favorable results [48]. Future experiments in vivo are needed in AR+-TNBC mice models to test our hypothesis under more physiological tumor conditions [48]. In Figure 3 we show that RUNX1 transcriptional activity inhibition has a strong negative effect on *KLF4* and *OCT4* gene expression. Both genes have been described as determinant factors involved in CSC development [49] and TNBC CSC enrichment [50,51]. Since four RUNX1 binding sites were identified in the *KLF4* promoter in a human leukemia cell model [52] and *KLF4* has been determined as an AR gene target in breast cancer cell lines [53], more experiments are needed to define the principal source of *KLF4* gene expression activation.

Furthermore, in Figure 4 we report that RUNX1 binds to the *SOX4* gene promoter and regulates its expression in standard and suspended conditions, suggesting that SOX4 could be one of the RUNX1 mediators in promoting CSC and/or chemoresistance in our model. In line with this, it has been described that stable overexpression of SOX4 in immortalized, non-transformed RWPE-1 prostate cells enables anchorage-independent growth and colony formation in soft agar [54]. It has recently been demonstrated that combined inhibition of Wnt signaling and SOX4 inhibits proliferation and migration and induces apoptosis of TNBC BT-549 cells [55]. In supplementary Figure S2C we show that inhibition of RUNX1 also potentiates Px and Enza toxicity in this AR^+^-TNBC cell line. Several studies have indicated that SOX4 also plays a critical role in EMT regulation, which can facilitate metastasis and chemoresistance in carcinomas [56]. Indeed, other groups have shown that SOX4 overexpression induces EMT in breast cancer cells [40], which in turn upregulates stem cell markers and enhances mammosphere formation [57]. In addition, SOX4 involvement in drug resistance has been described in colon and cervical cancer [42,58]. The present observation that RUNX1 regulates SOX4 unravels a potential mechanism by which RUNX1 regulates EMT in TNBC cell lines, previously reported in Ran (2020) [24]. In sum, our data strongly suggests that RUNX1 may be required for the enrichment of CSC in AR^+^-TNBC cell lines.

In contrast to TNBC, *RUNX1* and *CBFβ* have been described as tumor suppressor genes involved in reduced tumor growth and impaired EMT and CSC generation in ER^+^ breast cancer [27,59]. Remarkably, cumulative evidence supports the concept that the role of RUNX1 in breast cancer depends on the hormone receptor context [18,21,22,23,24].

Collectively, our results reveal that reduction of RUNX1 transcriptional activity significantly increases sensitivity to chemotherapy in AR^+^-TNBC cell lines, in both standard culture conditions and forced suspension. Pharmacologic inhibition of RUNX1 significantly enhances the previously described combination treatments, such as Px and Enza. Remarkably, we observed that cells grown in suspended conditions (CSC-like phenotype) need higher concentrations of drugs than those in attached conditions to generate a similar toxic effect. This observation is supported by accumulated evidence suggesting that CSC are the remaining population that survives drug treatments and regenerates tumors [49]. This is reinforced by Supplemental Figure S1A, where enzalutamide was not able to efficiently reduce AR binding levels in the *RUNX1* gene as in attached conditions (Figure 1A). Future analyses are necessary to determine the relative functional contributions of each AR binding region. RUNX1 participation in chemotherapy drug response has also been reported in ovarian cancer [60], glioblastoma multiforme [61] and colorectal cancer [62], suggesting that this function could be a general molecular mechanism of action that favors tumor aggressiveness against cytostatic drug treatment. In vivo experiments have yet to be performed to continue exploring this concept in TNBC.

Finally, it has been described that the tumor microenvironment is relevant in TNBC progression and chemoresistance [63]. Recently, Halperin (2022) reported that RUNX1 expression is upregulated in cancer-associated fibroblasts and that the RUNX1 signature is associated with poor breast cancer outcomes [64]. This recent evidence suggests that blocking the transcriptional activity of RUNX1 in vivo could simultaneously reduce tumor growth and increase its sensitivity to drugs, as well as weaken the pro-tumorigenic effect of the microenvironment.

## Data Availability

Not applicable.

## Supplementary Materials

The following supporting information can be downloaded at: https://www.mdpi.com/article/10.3390/cells12030444/s1.
