# Supplementary material for: RUNX1 Is Regulated by Androgen Receptor to Promote Cancer Stem Markers and Chemotherapy Resistance in Triple Negative Breast Cancer
Source: Cells. 2023 Jan 29;12(3):444. doi: 10.3390/cells12030444 (PMC9913961; doi:10.3390/cells12030444)
Supplement: Supplementary file 1 [file cells-12-00444-s001.zip › OK_R2_cells-2075422 supplementary revised.pdf]

Supplementary Figure S1

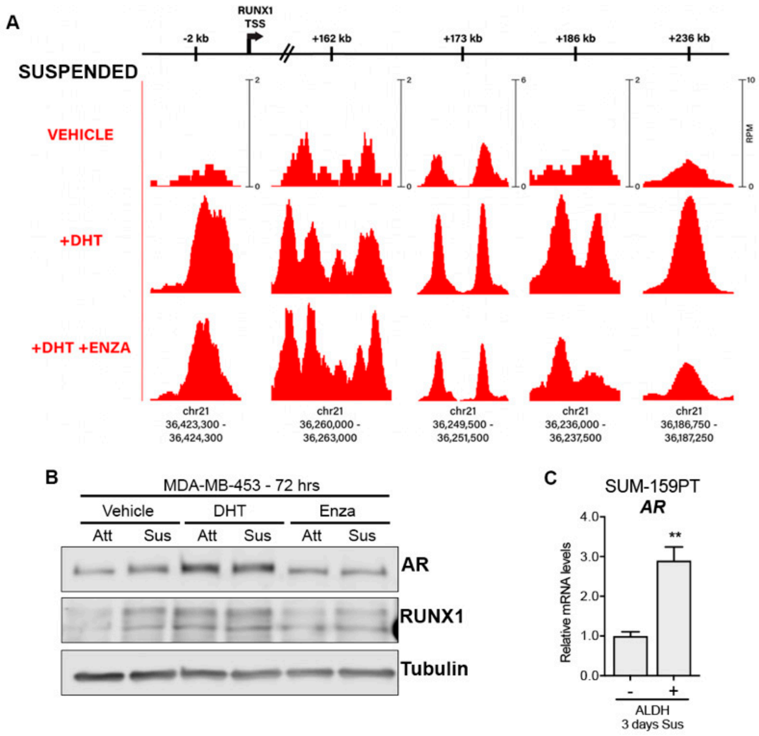

Supplementary Figure S1. A) MDA-MB-453 cells were cultured in forced suspended conditions and treated with either vehicle (DMSO+ethanol), DHT (10 nM) or DHT+Enza (20 µM) for 24 hrs. AR ChIP-seq was performed and the *RUNX1* promoter and intronic regions were analyzed. The scale bars at the top are labeled for each locus to indicate the relative distance from the canonical *RUNX1* transcription start site (TSS). The AR ChIP-seq signals are shown for each treatment condition at multiple loci within RUNX1. The scale at each position (shown in the 'VEHICLE' track) indicates the normalized range of signal values within that region (as measured by reads per million, RPM) and is the same for all treatment conditions to allow for accurate comparison of the peak intensities. The chromosomal coordinates of each peak are shown below. B) AR and RUNX1 Western blot of MDA-MB-453 cells in attached (Att) or forced-suspended conditions (Sus) and treated with either vehicle (DMSO+ethanol), DHT (10 nM) or Enza (20 µM) for 72 hrs. Tubulin was used as a housekeeping control. C) SUM-159PT were cultured in forced-suspended conditions (Sus) for 3 days and then were sorted using the ALDEFLUOR assay. mRNA was prepared from $ALDH^-$ and $ALDH^+$ subpopulations and AR levels were evaluated by qPCR. One representative experiment is shown in B. Student t-test was performed in C from three independent experiments., ** p<0.01.

Supplementary Figure S2

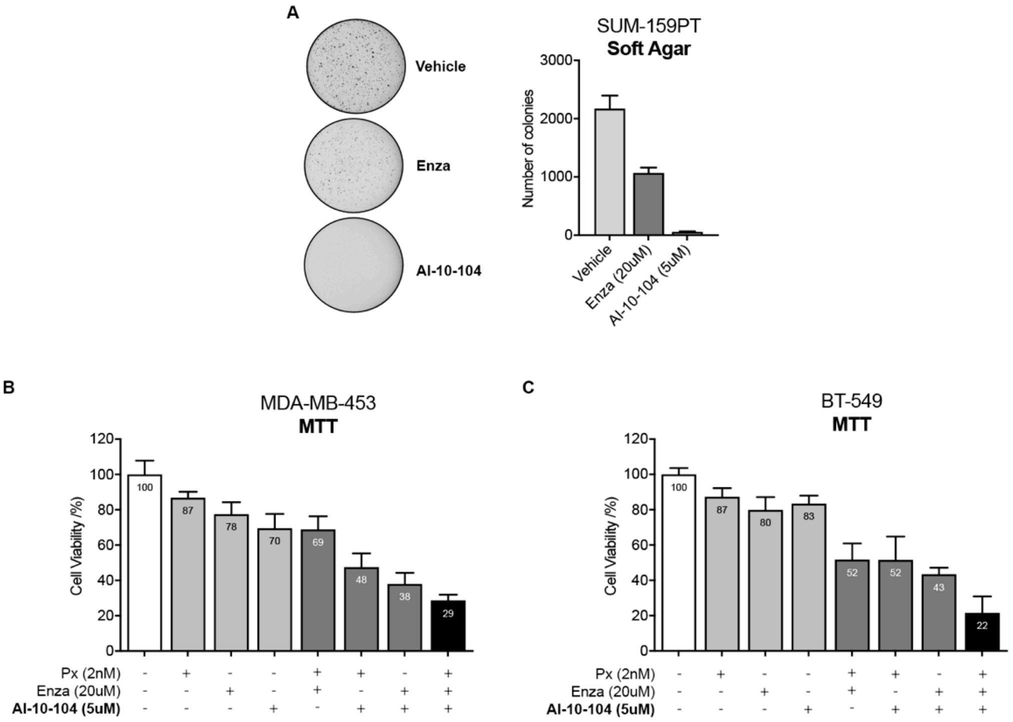

Supplementary Figure S2. A) SUM-159PT were cultured in soft agar conditions for 21 days. Crystal violet staining was performed, and the number of colonies were counted. The left panel shows a representative image of each treatment and the right panel the quantification and standard deviation. B, C) MTT assays were performed in MDA-MB-453 (B) and BT-549 (C) treated with 2 nM Paclitaxel (Px), 20 μM Enzalutamide (Enza), 5 μM AI-10-104 or all the combinations for 72 hrs. Results are expressed as the percentage of cell viability relative to control treatment (DMSO). One representative experiment of two is shown in B and C,
